# Proteome analysis of serovars Typhimurium and Pullorum of *Salmonella enterica *subspecies I

**DOI:** 10.1186/1471-2180-5-42

**Published:** 2005-07-18

**Authors:** Vesela Encheva, Robin Wait, Saheer E Gharbia, Shajna Begum, Haroun N Shah

**Affiliations:** 1 Molecular Identification Services Unit, NCTC, Centre for Infections, Health Protection Agency, London, UK; 2 Genomics Proteomics and Bioinformatics Unit, Centre for Infection, Health Protection Agency, London, UK; 3 Kennedy Institute of Rheumatology Division, Faculty of Medicine, Imperial College London, UK

## Abstract

**Background:**

*Salmonella enterica *subspecies I includes several closely related serovars which differ in host ranges and ability to cause disease. The basis for the diversity in host range and pathogenic potential of the serovars is not well understood, and it is not known how host-restricted variants appeared and what factors were lost or acquired during adaptations to a specific environment. Differences apparent from the genomic data do not necessarily correspond to functional proteins and more importantly differential regulation of otherwise identical gene content may play a role in the diverse phenotypes of the serovars of *Salmonella*.

**Results:**

In this study a comparative analysis of the cytosolic proteins of serovars Typhimurium and Pullorum was performed using two-dimensional gel electrophoresis and the proteins of interest were identified using mass spectrometry. An annotated reference map was created for serovar Typhimurium containing 233 entries, which included many metabolic enzymes, ribosomal proteins, chaperones and many other proteins characteristic for the growing cell. The comparative analysis of the two serovars revealed a high degree of variation amongst isolates obtained from different sources and, in some cases, the variation was greater between isolates of the same serovar than between isolates with different sero-specificity. However, several serovar-specific proteins, including intermediates in sulphate utilisation and cysteine synthesis, were also found despite the fact that the genes encoding those proteins are present in the genomes of both serovars.

**Conclusion:**

Current microbial proteomics are generally based on the use of a single reference or type strain of a species. This study has shown the importance of incorporating a large number of strains of a species, as the diversity of the proteome in the microbial population appears to be significantly greater than expected. The characterisation of a diverse selection of strains revealed parts of the proteome of *S. enterica *that alter their expression while others remain stable and allowed for the identification of serovar-specific factors that have so far remained undetected by other methods.

## Background

Enteric bacteria classified as *Salmonella *are responsible for a wide variety of illnesses, including typhoid fever, food poisoning, gastroenteritis and septicaemia. The genus *Salmonella *comprises two species, namely *Salmonella enterica *which can be subdivided into more than 2 300 serovars, and *Salmonella bongori*. It has been proposed that the evolution of the genus has progressed in three major phases [1]. In the first phase *Salmonella *diverged from *E. coli *by acquiring *Salmonella *pathogenicity island 1 (SPI 1) which encodes virulence factors required by *Salmonella *for the intestinal phase of the infection [2]. The formation of the two species *S. enterica *and *S. bongori *is considered the second phase in the evolution in which *Salmonella *pathogenicity island 2 (SPI 2) was acquired. Its role in pathogenicity is yet to be established but its relevance has been demonstrated for the development of systemic infection [3]. The last stage of the evolution of *Salmonella *is considered to be the formation of *S. enterica *subspecies I, which enabled a dramatic expansion of host specificity of the species. While the rest of the subspecies of *S. enterica *are adapted to heterothermic vertebrates, subspecies I strains are capable of colonising mammalian and avian hosts [4].

*S. enterica *subspecies I is routinely subdivided into serovars on the basis of the expression of three surface antigens (Ag's), the somatic O Ag, the flagella H1 and H2 Ags, and the capsular Vi Ag, according to the Kauffmann-White scheme [5]. Although the serovars are very closely related, they have different host ranges and cause different disease signs. Serovar Typhimurium is a generalist that infects a wide range of animals (humans, wild rodents, poultry, pigs, cattle). Some serovars are host specific, infecting only one animal host *e.g*. serovar Pullorum infects only poultry and serovar Typhi infects only humans [4].

The genetic and molecular basis for the different host ranges and host specificities of the serovars of *S. enterica *subspecies I are not clear. The evolution and acquisition of the pathogenicity islands of *Salmonella *leading to the formation of subspecies I and allowing for the use of homeothermic animals as a host is extensively studied. However, it is not clear how host-restricted serovars appeared and whether they acquired different virulence determinants compared to the host generalists.

For serovar Pullorum the process of host adaptation has been accompanied by point mutations resulting in loss of the ability to mediate mannose sensitive agglutination (MHSA) and to express flagella [6,7]. Strains of serovars Typhimurium isolated from avian hosts also lack mobility and MHSA [4], and these changes also result in a 100-fold reduction in the virulence of serovar Typhimurium in mice [7]. However, the highly host-specific phenotype of serovar Pullorum cannot be explained by these point mutations alone. For instance, serovar Typhimurium initiates disease by entering the Peyer's patches, where it invades the circulating lymphoid cells [8]. In contrast, serovar Pullorum is incapable of entering the Peyer's patches, cannot survive and multiply in the cells of the mouse reticuloendothelial system, and is internalised by the murine macrophages by a mechanism different to the one of serovar Typhimurium [9-11].

Initial hybridisation studies showed that the serovars of *S. enterica *share >90 % of their DNA content [12]. The comparison of their genomes revealed that despite their similarity each serovar has many insertions and deletions relative to the other serovars, which vary in size from 1 to 50 kB [13]. However, the differences observed at the DNA level have so far not been related to protein expression. It is of great importance to determine if the differences observed at the genomic level are in fact translated into proteins as has been reported by Taoka and co-workers [14], who found that the majority of the horizontally transferred genes in the genome of *E. coli *are not translated into proteins, presumably because they are inadequate for the translational machinery of the cell.

Another approach for the identification of serovar-specific factors involved the introduction of virulence-associated DNA regions of host generalists into host-specific serovars, to expand their host range. This approach was unsuccessful, suggesting that multiple genes are responsible for the host-restricted phenotypes [15]. Therefore, scientific approaches which enable global measurements of gene expression on a genome-wide scale would give a better understanding of the differences in gene-regulation patterns between serovars with host restricted and host-generalist phenotypes.

In this study we used a standard proteomic approach combining two dimensional gel electrophoresis (2D GE) and tandem mass spectrometry (LC/MS/MS) to compare the expression patterns of isolates of the highly host-restricted serovar Pullorum and the host generalist serovar Typhimurium. The protein expression patterns of *S. enterica *serovar Typhimurium have been extensively studied and an annotated reference map of the cell envelope proteins of serovar Typhimurium has been published [16,17]. Over 800 proteins expressed by serovar Typhimurium have been recently identified using two-dimensional HPLC-MS, including some potentially associated with multiple antibiotic resistance [18]. Changes in the expression pattern of serovar Typhimurium have been monitored when grown in media with low pH [29,30] and when exposed to bile [21], and an attempt has been made to identify proteins with increased levels of expression when grown intracellularly [22]. However, the protein expression pattern of serovar Pullorum is yet to be determined and, more importantly, comparative analysis of strains with different sero-specificity have not been performed to date. A comparison of the proteome of a serovar that is not host-specific to the serovar that is highly host-specific may help reveal what factors enabled *Salmonella *to overcome species barriers and adapt to new hosts and, ultimately, should give an insight into the process of host adaptation and the emergence of new pathogens.

## Results and discussion

An annotated reference map of *S. enterica *cytosolic proteins was created using strain 74 of serovar Typhimurium obtained from the National Collection of Type Cultures (London, UK). Seven hundred and seventy-one spots were detectable after staining with SYPRO Ruby, of which 233 were identified by LC/MS/MS. The proteins identified represented 200 open reading frames (ORF's), which constitute 4.4 % of the 4558 protein coding sequences predicted in the genome of Salmonella Typhimurium LT2 [23].

Each protein was represented, on average, by 1.165 spots on the gel. Twenty-four proteins were detected in more than one isoelectric form. Aldehyde dehydrogenase B, glyceraldehyde-3-phosphate dehydrogenase and an oligopeptide-binding protein precursor were each present in four isoforms. For most of these the pI difference was relatively small, but differences of around two to three pI units were also observed. In the case of transaldolase A and thiol disulphate, interchange protein forms were observed which differed in molecular mass as well as pI. In the reference map of *Salmonella *many of the substrate-binding proteins identified appeared as a series of isoelectric forms, which was indicative of posttranslational modifications or amino acid substitutions. Such diversity of isoforms has been described for other bacterial proteomes. The ratio of the number of spots to the number of ORFs has been reported to be 1.4–2 in *E. coli *[24,25], 1.6 for *Chlamydia pneumoniae *[26] and 1.42 for *Staphylococcus aureus *[27]. It should be noted that, in most cases, the multiplicity of the pI exhibited by a protein was not accompanied by a significant change in the molecular weight, suggesting that the generation of isoelectric forms may involve modifications such as phosphorylation [28], methylation [29,30] or deamidation [31], rather than an introduction of high molecular weight groups such as long glycan chains. Some of the isoforms observed appeared to be serovar-specific. The isoforms of the enzyme superoxide dismutase A differed in their pI point but had identical molecular weights. One of the isoforms was expressed by all isolates of serovar Typhimurium and the second isoform was characteristic for serovar Pullorum. Similarly, serovar Pullorum expressed three different forms of the lysine, arginine, ornithine-binding periplasmic protein precursor while the expression maps of the isolates of Typhimurium contained only two isoforms. However, the exact nature of these putative posttranslational modifications and their physiological relevance remains to be elucidated, and the possibility that some isoforms occur during sample preparation cannot be ruled out.

Some of the most intense spots on the gels were attributable to metabolic proteins, including the full set of Krebs cycle enzymes and all the enzymes of glycolysis with the exception of glucokinase, which catalyses the first step of the glycolytic pathway. The gene for glucokinase is present in the genome of *Salmonella *Typhimurium and as a glycolytic enzyme it is localised in the cytosol. The predicted molecular weight and pI point from the genome sequence is 34 564 Da and 5.83 respectively. Therefore theoretically the enzyme should be detected using the 2D GE protocol described. The failure to detect this enzyme may be due to its low expression or, possibly, because *S. enterica *obtains glucose 6-phosphate via a different route. A membrane bound enzyme complex, namely phosphoenol pyruvate phosphotransferase system (PTS), which couples the transport of sugars through the cell membrane with their phosphorylation, has been extensively studied in *E. coli *and *Salmonella *[32]. Two of the components of this system, enzyme I and a glucose-specific component IIA, were identified in the profile of serovar Typhimurium (spot 123 and 46, respectively). It is also possible that posttranslational modifications could have altered the pI of the glucokinase to a value outside the pH range of the IPG strip used.

Several of the enzymes of the pentose phosphate pathway were also identified, including glucose 6-phosphate dehydrogenase, transaldolase, transketolase and phosphogluconate dehydrogenase, which suggests that this pathway is also active under these growth conditions. Glucose 6-phosphate dehydrogenase also participates in the Entner Duodoroff pathway. However, since no other components of this pathway were detected it was likely that the pathway was repressed while glycolysis via the Embden Meyerhof pathway was active.

Many of the proteins identified were associated with protein biosynthesis, including elongation factors Tu, Ts, G and P, and tRNA synthetases for arginine, asparagine, aspartic acid, proline, glutamine, glycine, histidine, lysine and tyrosine. Chaperone proteins identified included dnaK, GroL, GroS and HtpG. Five ribosomal proteins (30S ribosomal protein S6, S1, S2 and 50S ribosomal proteins L7/L2, L9/L12) were detected, but others remained unobserved, presumably because of their high basicity [27].

An important subset of the proteins detected are involved in defence against oxidative damage, including two superoxide dismutases (SodA and SodF), alkyl hydroperoxide reductase (22 kDa subunit), a putative peroxidase, an oxidoreductase (ucpA), a putative catalase and catalase HPI. Under anaerobic conditions expression levels of SodA and the alkyl hydroperoxide reductase were reduced, whereas SodF was upregulated (data not shown). We also identified thirteen hypothetical proteins for which no function has yet been assigned.

### Comparative analysis of the expression patterns of serovars Typhimurium and Pullorum

Comparison of the expression maps of serovars Typhimurium and Pullorum revealed that, despite the similarities in the expression patterns there was a high degree of variation amongst clinical and laboratory isolates, even when they were from the same serovar. This finding corresponds to the comparative analysis of the serovars of *Salmonella *performed using a DNA microarray [33], which indicated that classification of *Salmonella *strains into genomovars based on the similarity in their genome sequences is more appropriate but does not always correspond to serovar.

A similar selection of proteins were expressed by all isolates and differences observed were mainly in the level of expression but a few characteristic proteins were also present. The comparison of the expression patterns revealed that there was no variation in expression of enzymes involved in glycolysis, Krebs cycle and the pentose phosphate pathway, as well as the chaperones and the proteins involved in protein biosynthesis. The majority of the differences observed were not serovar-specific *e.g *the laboratory reference strain of serovar Typhimurium overexpressed several substrate-binding periplasmic proteins including maltose binding protein precursor, oligopeptide binding protein precursor and ABC superfamily dipeptide transport protein, while the clinical isolates of the same serovar expressed these proteins at very low levels. Such differences may be due to the different conditions that the laboratory strains and clinical isolates are subjected to, with the former gradually adapting to the laboratory while undergoing continuous subculturing. These may have favoured changes which otherwise would not have happened in non-laboratory conditions. A similar observation was reported for *Helicobacter pylori *by Hynes and co-workers [34]. Therefore it is becoming increasingly apparent that the expression profiles of both laboratory reference strains and clinical isolates should be considered when characterising the proteome of any microbial pathogen [35].

Despite the high level of variation amongst the isolates characterised, several serovar-specific factors were also observed. Two transport proteins, sulphate (sbp) and thiosulphate (cysP) binding proteins, were detected as prominent spots in the profile of serovar Pullorum but were not expressed by any of the Typhimurium isolates (Figure 3). Several strains representing other serovars (Enteritidis, Choleraesius and Dublin) were also tested and none of them expressed these two transport proteins, suggesting that under the growth conditions utilised they are characteristic only for serovar Pullorum. Furthermore, serovar Pullorum showed a significantly higher (p = 1.618 × 10^-4^) level of expression of the enzyme cysteine synthase (cysK) in comparison to serovar Typhimurium (Figure 3).

It has been reported that *E. coli *and *S. enterica *serovar Typhimurium harbour a sulphate transport system which is part of the cysteine regulon and is controlled in parallel with cysteine-biosynthetic enzymes [36]. CysP is part of this system and its expression in serovar Typhimurium is induced when grown under sulphur limitation [37]. However, it is possible that in serovar Pullorum this protein is constitutively expressed. Although the sulphate binding protein (Sbp) has been extensively studied [37-39] its functional relationship with cysP is yet to be elucidated. Their increased level of expression correlated with the increased level of cysteine synthase (CysK), which is controlled by the same operon. It is likely therefore that there is a functional association between these three proteins which may be relevant to the host adaptation of serovar Pullorum.

Another difference in the expression patterns was the absence of the protein yghA in the expression map of serovar Pullorum. YghA has been annotated as a putative oxidoreductase but its function is not clear. The characterisation of the genome of serovar Pullorum revealed a high level of genomic plasticity caused by a large insertion disrupting the balance of the genome [40]. Further studies should be aimed at determining if the gene for this hypothetical protein is present in the genome of serovar Pullorum or if the difference in the expression of yghA is a result of differential regulation on translational or post-translational level.

## Conclusion

Microorganisms vary in their mechanisms of survival, some remaining at a particular site where nutrients are more favourable, while others are metabolically more versatile and disseminate more readily and consequently manifest different disease signs. Such differences in the capacity to spread and adapt to different conditions can be observed amongst the serovars of *Salmonella enterica *subspecies I [41,42]. The existence of host generalist and host-adapted variants of this species presents a unique opportunity to study the mechanisms defining the process of host adaptation.

During adaptation to a new environment the metabolic activity of the cell can be expected to change. This was confirmed by the differential expression of the two substrate transport proteins (sulphate and thiosulphate binding protein) which were characteristic for the host-adapted serovar Pullorum. It can be speculated that the high sulphurous content of the egg, has favoured the increased expression of sulphate transporting proteins in serovar Pullorum. The sulphate ions may subsequently be reduced and used for the synthesis of cysteine [43] which corresponds to the elevated level of expression of the enzyme cysteine synthase reported in this study.

The differences observed between serovars Typhimurium and Pullorum suggest that there are variations in the expression patterns even between closely related bacteria. In this case 2D GE combined with MS proved a useful tool for identifying proteins differentially expressed in serovars with different host specificity and pathogenic potential. Future studies are now in progress to compare the proteome of a large number of serovars that specifically affect man.

## Methods

### Bacterial strains

The profiles of seven independent isolates of serovar Typhimurium and two of serovar Pullorum were used for the compative analysis. Strain 74 of serovar Typhimurium and 10704 of Pullorum were obtained from the National Collection of Type Cultures, (Health Protection Agency, London, UK). Strains A01, C01 of serovar Typhimurium and B52 of serovar Pullorum were obtained from the *Salmonella *Reference Collection at the University of Calgary, Canada. Four additional clinical isolates of serovar Typhimurium (strains 23, 56, 204 and 227) were supplied by the Department of Risk Research in VLA-Weybridge, UK.

### Protein extraction

Bacteria were cultured on Columbia Blood Agar (Oxoid, Basingstoke, UK) for 18 h at 37°C under standardised conditions. Cells harvested from three plates were lysed in 100 μl of 0.3% w/v SDS, 200 mM DTT and 50 mM Tris (pH = 8.0), then digested with DNase I and RNase A. The samples were vortexed with 0.3 g of glass beads (< 105 μm diameter) and homogenised five times for 1 min using a Mickle cell disintegrator (Mickle Laboratory Engineering Co. Ltd, UK) with 1 min cooling on ice after each homogenisation period. Cell debris was removed by centrifugation (21 000 × g for 30 min at 4°C) and proteins were mixed with urea/thiourea based rehydration solution [44].

### 2D GE

Samples containing a total of 150 μg of protein were loaded into 18 cm IPG strips (pH 3–10 NL, Amersham Biosciences, UK) by in-gel rehydration [45]. IEF was performed using an Investigator 5000 apparatus (Genomic Solutions, USA) for 24 h, (85 000 Vh at a maximum voltage of 5000 V and a maximum current of 110 μA.)

The focused IPG strips were equilibrated twice (2 × 30 min) in DTT and iodoacetamide as described by Görg *et al*., [46]. Second dimension electrophoresis was performed on 10 % Duracryl^® ^gels (Proteomic Solutions, France) using Tris/Tricine buffer chemistry as recommended by Fountoulakis *et al*., [47], for 5 h, at a maximum voltage of 500 V and maximum power of 20 000 mW per gel.

Spots were visualized with SYPRO Ruby^® ^(Molecular Probes, UK) then counter-stained with colloidal Coomassie G [48] prior to manual spot excision.

### Reproducibility and data analysis

To perform the comparative analysis a total of twelve profiles of serovar Typhimurium were analysed, including four replicates of the profile of strain 74 and duplicate profiles of strains 204 and 227 and additional profiles of strains 23, 56, A01 and C01. Five profiles of serovar Pullorum were used for the comparison, three of strain 10704 and two of strain B52.

The intensity values of the protein of interest were estimated using ProteomWeaver software (Definiens, Germany) and the values were subjected to the Students T-test. Differential expression was reported only when the intensity values were found significantly different (p < 0.05).

### Trypsin digestion and LC/MS/MS

Excised spots were digested *in situ *with trypsin, using an Investigator ProGest robotic digestion system (Genomic Solutions, Huntington, UK) as previously described [49]. Tandem electrospray mass spectra were recorded using a Q-TOF hybrid quadrupole / orthogonal acceleration time of flight spectrometer (Micromass, Manchester, UK) interfaced to a Micromass CapLC capillary chromatograph. Samples were dissolved in 0.1% v/v formic acid, and injected onto a Pepmap C18 column (300 μm × 0.5 cm; LC Packings, Netherlands), The capillary voltage was set to 3,500 V, and data-dependent MS/MS acquisitions were performed on precursors with charge states of 2, 3 or 4 over a survey mass range of 500–1300. The collision voltage was varied between 18 and 45 V depending on the charge and mass of the precursor.

### Database searching parameters

Proteins were identified by correlation of uninterpreted tandem mass spectra to entries in SwissProt/TREMBL, using ProteinLynx Global Server (Versions 1.1, Micromass). No taxonomic, mass or pI constraints were applied. One missed cleavage per peptide was allowed, and the fragment ion tolerance window was set to 100 ppm. Carbamidomethylation of cysteine was assumed, but other potential modifications were not considered in the first pass search. All matching spectra were reviewed manually, and in cases were the score reported by ProteinLynx global server was less than 100, additional searches were performed against the NCBI nr database using MASCOT, which utilizes a robust probalistic scoring algorithm [50]. Where identifications were based on a single matching peptide the sequences were confirmed by manual sequencing using the MassLynx program Pepseq. Measured parent and fragment masses were typically within 0.03 Da of their calculated values.

## Abbreviations

Ag antigen

DTT dithiothreitol

HPLC high performance liquid chromatography

IEF isoelectric focusing

IPG immobilised pH gradient

LC liquid chromatography

MS/MS tandem mass spectrometry

SDS sodium dodecyl sulphate

SPI *Salmonella *pathogenicity island

ORF open reading frame

Tris tris (hydroxymethyl) aminomethane

2D GE two dimensional gel electrophoresis

## Authors' contributions

VE carried out the protein work, analysed the 2D GE profiles and drafted the manuscript. RW and SB performed the mass spectrometry identification of the proteins. VE, HNS and SEG participated in the design of the study. HNS conceived and coordinated the study. All authors read and approved the final manuscript.

## Supplementary Material

**Figure 1:**
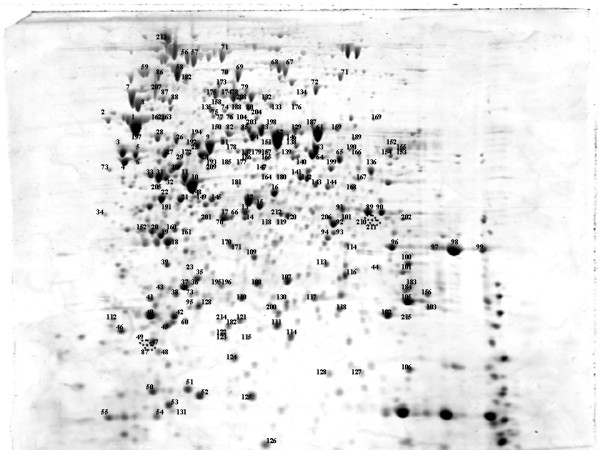
Annotated reference map of the cytosolic proteins of *S. enterica *serovar Typhimurium. Separation was performed with 18 cm pH 3–10 NL IPG strips and 10 % Duracryl gels. Spots were visualised with SYPRO Ruby and imaged with a Typhoon Scanner (Amersham Biosciences, UK). The labelled spots were excised and analysed by LC/MS/MS, (Additional file 1). The empty circles represent spots detected only in the profile of serovar Pullorum.

**Figure 3:**
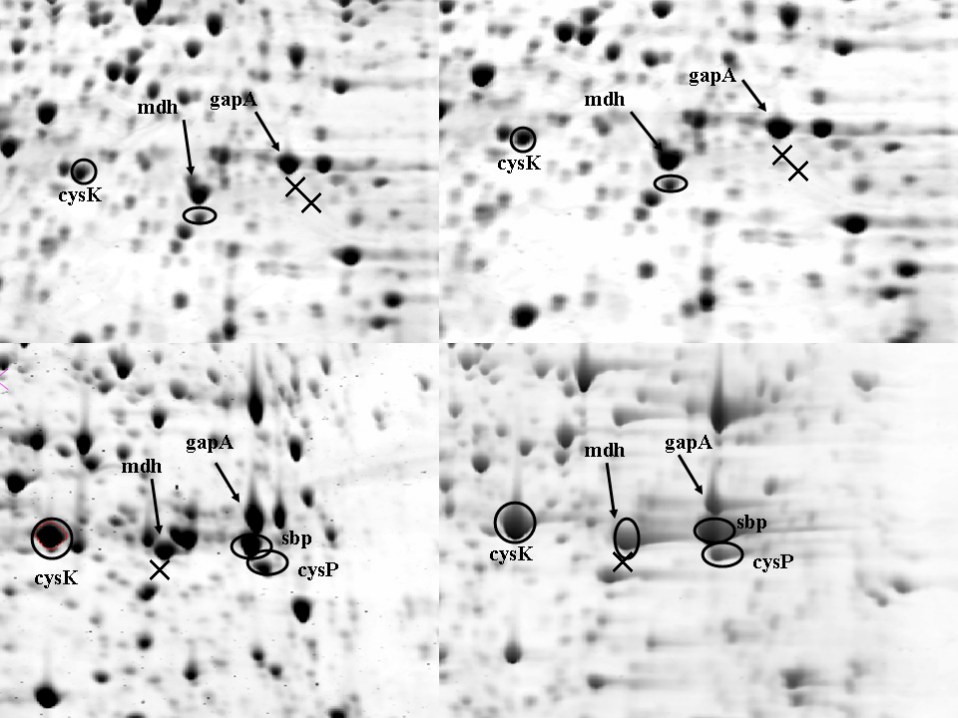
Comparison of the protein expression of serovars Typhimurium (A and B) and Pullorum (C and D). The strains of serovar Pullorum expressed sulphate (Sbp) and thiosulphate binding protein (cysP) which were absent from the profile of Typhimurium. The enzyme cysteine synthase (cysK) was detected in the profile of both serovars but showed two fold higher expression in serovar Pullorum. The hypothetical oxidoreductase yghA was present in the profiles of serovar Typhimurium but absent from Pullorum. Mdh and gapA were present in all profiles and were used as reference spots in this comparison. The (x) symbols correspond to protein spots missing from the corresponding area of the gel.

Additional File 1 Protein identities in the reference map of Salmonella Typhimurium obtained using LC/MS/MS. The table contains the list of proteins identified in the reference map of Salmonella Typhimurium accompanied by their spot number on the gel image (Figure 1) and by their unique SWISS Prot identifier. The amino acid sequences of the matching peptides determined using LC/MS/MS are also included. Click here for file

## Figures and Tables

**Figure 2:**
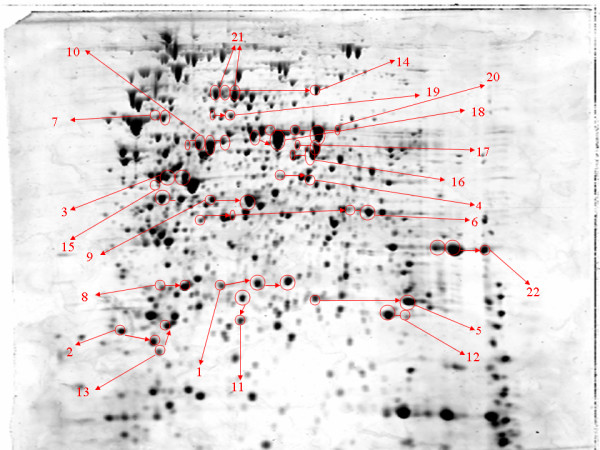
Annotated map of the cytosolic proteins of *S. enterica *expressed in more than one mobility form (isoform). A total of 21 proteins were detected as more than one spot. The identities of the proteins are presented in (Additional file 1). Each protein spot is outlined with a red circle and the isoforms are connected with red arrows. The higher number of isoforms (4), were detected for aldehyde dehydrogenase B (spot 10), oligopeptide binding protein precursor (spot 20) and glyceraldehyde 3-phosphate dehydrogenase (spot 6). The majority of the isoforms differed slightly in pI point but in some cases (e.g spot 13 and 11) differences in the molecular weight were also observed.

**Table 1:** Cytosolic proteins of serovar Typhimurium detected in more than one isoform.

Spot number	Protein	Number of isoforms	Gene name
1	2,3 bisphosphoglycerate dependent phosphoglycerate mutase	3	gpmA
2	DnaK suppressor protein	2	dksA
3	Enolase	2	eno
4	Glutamate dehydrogenase	2	gdhA
5	Glutamine binding periplasmic protein precursore	2	galU
6	Glyceraldehyde 3-phosphate dehydrogenase	4	gapA
7	Lysil tRNA synthetase	2	lysS
8	Lysil arginine ornithine binding periplasmic protein	3	argT
9	Maltose binding protein precursor	2	malE
10	Aldehyde dehydrogenase B	4	aldB
			
11	Superoxide dismutase F	2	sodF
12	Superoxide dismutase A	2	sodA
13	Thiol:disulphate interchange protein	2	dsbA
14	Transketolase	2	STY3236
15	Phosphoglycerate kinase	2	pgk
16	Putative NAD dependent aldehyde dehydrogenase	2	STM 1627
17	Dihydrolipoamide dehydrogenase	2	lpdA
18	ABC superfamily dipeptide transport protein	2	dppA
19	Silent usher protein precursor	2	ushA
20	Periplasmic oligopeptide binding protein precursor	4	oppA
			
21	Acetyl CoA synthase	2	acs
22	Glutamate aspartate binding periplasmic protein precursor	3	gltI
