# Supplementary material for: Proteome analysis of serovars Typhimurium and Pullorum of Salmonella enterica subspecies I
Source: BMC Microbiol. 2005 Jul 18;5:42. doi: 10.1186/1471-2180-5-42 (PMC1181816; doi:10.1186/1471-2180-5-42)
Supplement: Additional File 1 — Protein identities in the reference map of Salmonella Typhimurium obtained using LC/MS/MS. The table contains the list of proteins identified in the reference map of Salmonella Typhimurium accompanied by their spot number on the gel image (Figure 1) and by their unique SWISS Prot identifier. The amino acid sequences of the matching peptides determined using LC/MS/MS are also included. [file 1471-2180-5-42-S1.doc]

| **Spot number** | **Protein** | **SWISS PROT** **identifier** | **Unique sequences**  **matched** |
| --- | --- | --- | --- |
| **1** | 60 kDa chaperonin | CH60_SALTY | (R)VEDAL HATR(A)  (K)GQNED QNVGI K(V)  (R)GIDKA VAAAV EELK(A)  (K)AIAQV GTISA NSDET VGK(L)  (K)DTTTI IDGVG EEAAI QGR(V)  (R)QIVLN CGEEP SVVAN TVK(G)  (K)ANDAA GDGTT TATVL AQSII TEGLK(A) |
| **2** | N utilization substance | NUSA_SALTY | (K)EILAV VEAVS NEK(A)  (R)IEVPE IGEEV IEIK(A) |
| **3** | Phase 2 flagellin | FLJB_SALTY | (K)DDAAG QAIAN R(F)  (K)IDAAL AQVDA LR(S)  (R)FNSAI TNLGN TVNNL SEAR(S)  (-)AQVIN TNSLS LLTQN NLNK(S)  (R)NANDG ISIAQ TTEGA LNEIN NNLQR(V) |
| **4** | Phase 1 flagellin | FLIC_SALTY | (K)DDAAG QAIAN R(F)  (K)SQSAL GTAIE R(L)  (R)VSGQT QFNGV K(V)  (K)IDAAL AQVDT LR(S)  (R)FNSAI TNLGN TVNNL TSAR(S)  (-)AQVIN TNSLS LLTQN NLNK(S) |
| **5** | Trigger factor | AB0558 | (K)VRIDG FR(K)  NFVDALIK  NFVDALIKEK  ELPELTEEFIK  ELPELTEEFIKR  (K)NMERE LK(S)  (K)EVIEF YSK(N)  (R)ELFEE QAKR(R)  RFGVEDGSVAGLR  FGVEDGSVAGLR  INPAGAPNYVPGEYK  (R)VVVGL LLGEV IR(T)  (R)MIPGF EDGIK GHK(A)  MQVSVETTQGLGRR  ANDIDVPSALIDSEIDVLR  (R)FGGNE KQALE LPR(E)  (R)VTITI AADSI ETAVK(S)  (R)NVALE EQAVE AVLAK(A)  (R)RVTIT IAADS IETAV K(S)  (R)VTIDF TGSVD GEEFE GGK(A)  (K)AGEEF TIDVT FPEEY HAENL K(G) |
| **6** | ATP synthase beta subunit | Q8XGX4 | (R)YTLAG TEVSA LLGR(M)  (R)VYDAL EVQNG NEK(L)  (R)FLSQP FFVAE VFTGS PGK(Y) |
| **7** | Chaperon Hsp 70 | DNAK_SALTY | (R)LINYL VDEFK K(D)  (R)AKLES LVEDL VNR(S)  (R)QAVTN PQNTL FAIK(R)  (K)TAIES ALNAL ETALK GEDK(A) |
| **8** | 30S Ribosomal protein S1 | gi29142376 | (K)SESAI PAEQF K(N)  AVIESENSAER  (K)GATVE LADGV EGYLR(A) |
| **9** | Aldehyde dehydrogenase B | Q8ZL72 | (K)EGADI LTGGR(R)  (K)EGYYL EPTIL FGK(N) |
| **10** | Enolase | ENO_SALTY | (K)AVGAV NGPIA QAILG K(D)  (K)IQLVG DDLFV TNTK(I)  (K)FNQIG SLTET LAAIK(M)  (K)AFTSE EFTHF LEELT K(Q)  (K)VLGDK IQLVG DDLFV TNTK(I)  (R)SGETE DATIA DLAVG TAAGQ IK(T)  (K)GIANS ILIKF NQIGS LTETL AAIK(M) |
| **11** | Factor Tu | EFTU_SALTY | (R)TVGAG VVAK(V)  (K)FESEV YILSK(D)  (K)GYRPQ FYFR(T)  (R)HYAHV DCPGH ADYVK(N)  (K)MVVTL IHPIA MDDGL R(F)  (R)TKPHV NVGTI GHVDH GK(T)  (K)IIELA GFLDS YIPEP ER(A)  (R)HTPFF KGYRP QFYFR(T)  (R)AIDKP FLLPI EDVFS ISGR(G) |
| **12** | Maltose binding periplasmic protein precursor | MALE_SALTY | (K)KFEQD TGIK(V)  (K)AGLTF LIDMI K(N)  (K)LYPFT WDAVR(Y)  (K)VNYGV TLLPT FK(G)  (R)QTVDA ALKDA QSR(I)  (K)TWEEI PALDK ELK(V)  (R)FGGYA QSGLL AEVTP DK(A)  (K)LIAYP IAVEA LSLIY NK(D)  (K)LIAYP IAVEA LSLIY NKDLV PNPPK(T) |
| **13** | L-asparaginase | Q8XGY3 | (K)TNTTD VATFK(A)  (R)VLLQL ALTQT K(D)  (K)YGFVA SGTLN PQK(A)  (K)VGVEN LVDAV PQLK(D)  (K)AVNYG PLGYI HNGK(I)  (R)VPTGA TTQDA EVDDA(Y)  (K)VGIVY NYANA SDLPA K(A)  (K)TVFDT LATAA HNGTV VVR(S) |
| **14** | 6-Phosphofructokinase | GI/29143860 | (K)IGVLTS GGDAPG MNAAIR(G)  (R)YSVSD MINR (F)  (R)MGAYAI DLLLEGH GGR(C) |
| **15** | Alcohol dehydrogenase | Q8XG17 | (K)AAFNS AVDAV R(A)  (K)TGGAH AAVVT AVAK(A)  (K)VIAID VNDGQ LK(L)  (R)QDLTE AFQFA AEGK(V)  (K)AAVVT QDHQV DVTEK(T) |
| **16** | Fructose bisphosphate aldolase class II | Q8XFG7 | (K)IFDFV KPGVI TGDDV QK(V)  (K)ENEAYLQGQLGNPK(G)  (-)SKIFD FVKPG VITGD DVQK(V) |
| **17** | Glycerophosphodiester phosphodiesterase periplasmic precursor | Q8Z556 | (K)FTEGF DIENG K(K)  (K)AGVDG LFTDF PDK(A)  (R)YYAID FTLDE IK(S)  (K)QDNVY LQCFD VAELK R(I)  (R)IHTFE EEIEF VQGLN HSTGK(N)  (R)ADQLP DYATD VNQLY DILYN K(A) |
| **18** | D-galactose binding periplasmic protein precursor | DGAL_SALTY | (K)GEPGH PDAEA(T)  (K)ESGVI QGDLI AK(H)  (R)GQNVP VVFFN K(E)  (K)QNDQI DVLLA K(G)  (K)ALAIN LVDPA AAGIV IEK(A)  (K)SSIPV FGVDA LPEAL ALVK(S)  (R)VPYVG VDKDN LSEFT QK(-) |
| **19** | Elongation factor Ts | EFTS_SALTY | (K)AGNVA ADGVI K(T)  (K)DAGFQ AFADK(V)  (K)KAGNV AADGV IK(T)  (K)DAGFQ AFADK VLDAA VAGK(I) |
| **20** | Transaldolase B | TALB_SALTY | (R)ISTEV DAR(L)  (R)AQQVV DATDK(L)  (R)LSYDT EASIA K(A) |
| **21** | Succinyl-CoA synthase beta chain | SUCC_SALTY | (K)LVQQF TK(I)  (K)LGADG NALFR(Q)  (R)LEGNN AELGA K(K)  (K)AVLVN IFGGI VR(C)  (K)SLTDA AQQVV AAVEG K(-) |
| **22** | Phosphoglycerate kinase  Nucleoside permease | PGK_SALTY  Q815T0 | (R)ADLNV PVKEG K(V)  (R)VATEF SETAP ATLK(S)  (K)SLYEA DLVDE AKR(L)  (R)LLTTC DIPVP TDVR(V)  (K)DYLDG VDVAE GELVV LENVR(F)  (R)LVKDY LDGVD VAEGE LVVLE NVR(F)  (K)DQLSK IPK(H) |
| **23** | Septum site determining protein | Q8Z680 | (K)LVGVI PEDQS VLR(A) |
| **24** | Dihydrolipoamido succinyl transferase component of 2-oxoglutarat | Q8XFE6 | (R)GLVTP VLR(D)  (K)ELLED PTR(L)  (R)ESVGF LVTIK(E)  (K)APAVEPAAQPALGAR(G) |
| **25** | Aldehyde dehydrogenase B | Q8ZL72 | (K)EGADI LTGGR(R)  (R)ALVQE SIYER(F)  (R)IQPGE YGYPL K(L) |
| **26** | Aminoacyl-histidine dipeptidase | Q8ZRJ5 | (R)LIDFN GGTLR(N)  (R)LLNAT PNGVI R(N)  (K)TLVNA YQDIL K(N)  (K)TLVNA YQDIL KNELA EK(E) |
| **27** | Asparaginyl tRNA synthetase  Aspartate ammonia-lyase | SYN_SALTY  Q8XFR3 | (K)IYTFG PTFR(A)  (R)VAVDQ EVTVR(G)  (K)TFENP VFWGV DLSSE HER(Y)  (K)GEYQY LNPND HVNK(C) |
| **28** | Periplasmic trehalose binding protein precursor | TREA_SALTY | (R)NQSGF DLR(H)  (K)EYLPQ LQK(E)  (R)HFVDV NFTLP K(A)  (R)EHIDG LWPVL TR(S) |
| **29** | 6-phosphogluconate dehydrogenase | gi/146242 | EFVESLETPR  EKTEEVIAENPGQK |
| **30** | Isocitrate lyase  6-phosphogluconate dehydrogenase decarboxylating  Phosphopentomutase | ACEA_SALTY  6PGD_SALTY  DEOB_ECOLI | (R)THAGI EQAIS R(G)  (K)VLVPT QEAIQ(L)  (R)AAVLP ANLIQ AQR(D)  (K)IVSYA QGFSQ LR(A)  (R)KGPLNLPNLTR(L)  (K)ATGLDALFDATIK(E) |
| **31** | 3-oxoacyl [acyl carrier protein] synthase | FABB_ECOLI | (R)AVITGLG IVSSIGNNQQ EVLASLR(E) |
| **32** | Enolase | ENO_SALTY | (K)AVGAV NGPIA QAILG K(D)  (K)IQLVG DDLFV TNTK(I)  (K)VLGDK IQLVG DDLFV TNTK(I) |
| **33** | Isocitrate dehydrogenase | O30817 | (K)VVDAA VEK(A)  (K)GPLTT PVGGG IR(S)  (R)AAIEY AITND R(D) |
| **34** | Putative catalase | Q9KWV1 | (R)YFTQG LSDDD AGRR(E)  (K)GELAE GTENE AELYR(S) |
| **35** | Histidine binding periplasmic protein precursor | HISJ_SALTY | (K)KYFDF DVYGG(-)  (R)IGTDP TYAPF ESK(N)  (R)IDAAF QDEVA ASEGF LK(Q)  (K)NAQGE LVGFD IDLAK ELCK(R)  (R)IDAAF QDEVA ASEGF LKQPV GK(D) |
| **36** | Response regulator of two component regulatory system | Gi/16764586 | (K)GQGYLFEL(R)  (K)IQAQYPHDVITTVR(R)  (K)NSGLA SQVIN IPPFQ VDLS(R) |
| **37** | Lysine, arginine, ornitine binding periplasmic protein precursor | ARGT_SALTY | (K)EYAFA GPSVK(D)  (K)YFGDG TGVGL R(K)  (K)GSPIQ PTLES LK(G)  (K)GSPIQ PTLES LKGK(H)  (K)IADII SSLSI TDKR(Q)  (K)CTWVA SDFDA LIPSL K(A)  (R)LDAAL QDEVA ASEGF LKQPA GK(E) |
| **38** | Stringent starvation protein A, regulator of transcription | Q8Z3E9 | (R)DSFLA SLTEA ER(E)  (R)LPQLG IEFSG AGAK(E) |
| **39** | Oxidoreductase | UCPA_SALTY | (K)LADEL GGR(G)  (K)SLAVE YAQSG IR(V) |
| **40** | Osmotically inducible periplasmic protein  Alkyl hydroperoxide reductase c22 protein | Q8XET5  AHPC_SALTY | (K)LLADD LVPSR(K)  (K)STDIS VETNQ K(V)  (K)LLADD LVPSR K(V)  (K)GYAGD TATTS EVK(A)  (K)VVTLS GFVES QAQAE AAVK(V)  (K)EGEAT LAPSL DLVGK I(-) |
| **41** | Elongation factor P | EFP_SALTY | (K)VPLFV QIGEV IK(V) |
| **42** | Probable peroxidase  Putative periplasmic protein | Q8XGE8  Q8XET5 | (R)GSFLI DANGI VR(H)  (R)QAPDF TAAAV LGSGE IVDK(F)  (R) QAPDF TAAAV LGSGE IVDK NFK(N)  (K)VVTLS GFVES QAQAE AAVK(V) |
| **43** | Lysine, arginine, ornitine binding periplasmic protein precursor | ARGT_SALTY | (R)QQEIA FSDK(L)  (K)YFGDG TGVGL R(K)  (R)LDAAL QDEVA ASEGF LKQPA GK(E) |
| **44** | 30S ribosomal protein S2 | RS2_SALTY | (R)NKVHI INLEK(T)  (R)LKDLE TQSQD GTFEK(L) |
| **45** | Thiol disulphate interchange protein | DSBA_SALTY | (K)VTVPL FEAVQ K(T) |
| **46** | DnaK suppressor protein dksA  PTS system glucose specific IIA component | DKSA_SALTY  PTGA_SALTY | (R)AAQEE EFSLE LR(N)  (K)IVGDG IAIKP TGNK(M)  (K)LSGSV TVGET PVIR(I)  (K)VGDPV IEFDL PLLEE K(A)  (R)VKVGD PVIEF DLPLL EEK(E)  (K)DTGTI EIVAP LSGEI VNIED VPDVV FAEK(I) |
| **47** | DnaK suppressor protein  Probable thiol peroxidase | DKSA_SALTY  TPX_SALTY | (R)AAQEE EFSLE LR(N)  (K)DLSDV SLSQY AGK(V) |
| **48** | Thiol disulphate interchange protein | DSBA_SALTY | (K)TQTVQ SAADI RK(V) |
| **49** | Probable thiol peroxidase | TPX_SALTY | (K)DLSDV SLSQY AGK(R) |
| **50** | Osmotically inducible protein C  Ribosomal protein S6 | Q93RR7  RS6_SALTY | (K)VEAGF AITK(I)  (K)KGQAH WEGDI K(R)  (K)VAVAD IDAST FDQII QK(A)  (K)GTVST ESGVL NQQPY GFNTR(F)  (R)GKGTV STESG VLNQQ PYGFN TR(F)    (R)YSAAI TGAEG K(I) |
| **51** | DNA binding protein H-NS | HNS_SALTY | (-)SEALK ILNNI R(T)  (K)YSYVD ENGET K(T) |
| **52** | 10 kDa chaperonin | CH10_SALTY | (K)STRGE IIAVG K(G)  (K)SAGGI VLTGS AAGK(S)  (R)ILDNG TVQPL DVK(V)  (K)VGDIV IFNDG YGVK(S) |
| **53** | Universal stress protein | Q8ZLD7 | (K)HILIA VDLSP ESK(V) |
| **54** | Hypothetical protein | Q9X445 | (K)TGAVA EDVSA QAR(Q) |
| **55** | 50S ribosomal protein L7/L12 | RL7_SALTY | (K)TEFDV ILK(L)  (K)DLVES APAAL K(E)  (K)SLEEA GAEVE VK(-) |
| **56** | ClpB protein | Q8XFM5 | (K)VFVAE PSVED TIAIL R(G)  (R)GDNFI SSELF VLAAL ESR(G)  (K)RGDNF ISSEL FVLAA LESR(G) |
| **57** | Aconitate hydratase | Q8Z7D2 | (R)VALGD VPK(A)  (R)VVIAE SFER(I) |
| **58** | Elongation factor G | EFG_SALTY | (K)GIQEQ LK(S)  (K)GYEFI NDIK(G)  (K)SGPLA GYPVV DLGVR(L)  (K)IATDP FVGNL TFFR(V)  (R)VYSGV VNSGD TVLNS VK(T)  (R)EFNVE ANVGK PQVAY R(E)  (K)YLGGE ELTEE EIKQ LR(Q)  (R)INIID TPGHV DFTIE VER(S) |
| **59** | Phosphoenol pyruvate synthase | Q8Z6J0 | (R)DIFSL TNEEV QELAK(Q)  (R)DSGVV SELFD ERNDA VK(A)  (R)IYELL DKTDI DDVSQ LAK(A) |
| **60** | Hypothetical protein | Q9L435 | (R)DAALI AAAQK(V)  (K)TVEDL FIHLL SDTYS AEK(Q)  (K)VEHYE IASYG TLATL AEQLG YSK(A) |
| **61** | Periplasmic oligipeptide binding protein precursor | OPPA_SALTY | (K)APFND VR(V)  (K)KPATD LGVK(A)  (K)SPAFD KLIAD TLK(V)  (K)VADDT QRSEL YAK(A)  (K)TVINQ VTYLP ISSEV TDVNR(Y)  (K)ALDDH TFEVT LSEPV PYFYK(L)  (K)AEQQL DKDSA IVPVY YYVNA(R)  (K)TVINQ VTYLP ISSEV TDVNR YR(S)  (K)NNGSE VQSLD PHKIE GVPES NVSR(D) |
| **62** | ABC superfamily didpeptide transport protein | Q8Z299 | (K)AVYQG AGVAA K(N)  (K)IVTYE WGEYL K(R)  (K)ELNAD DVVFS FDR(Q)  (K)AFDGY WGTKP QIDR(L)  (K)WCYKP FEDLI QPAR(A)  (K)TGTTE VIPGL AEKWD ISEDG(T)  (K)GFTID LWAMP VQRPY NPNAR(R)  (K)AGTPE KVDLN PVGTG PFQLV QYQK(D) |
| **63** | Dihydrolipoamide dehydrogenase | Q8Z9E8 | (R)YDAVL VAIGR(V)  (R)VWDST DALEL K(E)  (K)GISYE TATFP WAASG R(A)  (K)VIPSI AYTEP EVAWV GLTEK(E) |
| **64** | Putative NAD dependent aldehyde dehydrogenase | Q8ZJZ3 | (R)VQGAA LTGSE K(A)  (R)VLAPN LAAGN PVLAK(H)  (K)IADQF LSQFT EAFRK(V)  (K)LANDS HYGLG GAVFS QNIER(A) |
| **65** | Fumarate hydratase class II | Q8Z6R6 | (K)VNQDL GLLAA EK(A)  (R)EHLIP QLSAL TDTLR(D) |
| **66** | Glyceraldehyde 3-phosphate dehydrogenase | G3PI_SALTY | (K)VGING FGR(I)  (K)AATYE QIK(A)  (K)KVVLT GPSK(D)  (K)AGIAL NDNFV K(L)  (R)VPTPN VSVVD LTVR(L)  (R) VPTPN VSVVD LTVR EK(A)  (K)GANFD KYEGQ DIVSN ASCTT NCLAP LAK(V) |
| **67** | Formate acetyl transferase | PFLB_ECOLI | (K)SGVLT GLPDA YGR(G) |
| **68** | Catalase HPII | Q8Z6H1 | (R)GPTLL EDFIL R(E)  (K)DPALS LYAVP DGDVK (G) |
| **69** | NADP dependent malic enzyme | MAO2_SALTY | (R)ISYNL LR(V)  (R)VVLPE GEEAR(V)  (K)IQVSP TKPLA TQR(G)  (K)QSALD FHEFP VPGK(I)  (K)AGVDF EIVNN ESDPR(F) |
| **70** | D-lactate dehydrogenase | Q8Z780 | (K)ELGLK VVR(V)  (K)SNDVI QDDVF R(R) |
| **71** | Fatty oxidation complex alpha subunit | FADB_SALTY | (R)ALVGI FLNDQ YVK(G)  (R)VLFPY FAGFS QLLR(D)  (K)KEEDA AVDDL LASVS QPK(R) |
| **72** | Transketolase II | Q874S9 | (R)QNLAQ VER(T)  (K)AYPEL AAEFT R(R)  (K)TPGHP EIGYT PGVET TTGPL GQGLA NAVGLAIAER(T) |
| **73** | Hypothetical protein | Q8XFP6 | (R)ALITQ DLSR(F)  (R)EGANV SIIEN GK(T) |
| **74** | ABC superfamily transport protein | P37797 | (K)VLEVS NLR(K)  (K)LASVD QFR(D)  (K)IGVLG LNGAG(S)  (K)VLEVS NLRK(S)  (R)VGELS GGERG R(L)  (K)NISLS FFPGA K(I)  (K)GAIVG IIGPN GAGK(S)  (R)LGDKV LEVSN LR(K)  (R)NETNE LFIPP GPR(L)  (K)RNETN ELFIP PGPR(L)  (K)LASVD QFRDS MDNSK(T)  (K)IGYLP QEPQL NPEHT VR(E) |
| **75** | Silent usher protein precursor | USHA_SALTY | (K)IAVIG LTTDD TAK(I) |
| **76** | Silent usher protein precursor | USHA)SALTY | (R)VILAA QIAR(T) |
| **77** | Arginyl tRNA synthetase | SYR_SALTY | (R)STIIG DAAVR(T)  (K)LADLL DEALE R(A)  (R)QTIVV DYSAP NVAK(E)  (K)ANIDE QALAS APVII SEDRE AQLAA R(L) |
| **78** | Acetyl CoA synthetase | Q8Z1R0 | (K)HAIPA NIADR(C)  (R)FANTL LDLGI K(K)  (R)VDDVL NVSGH R(L)  (K)ILDWI TPYQK(V)  (R)CLINP EQYET K(Y)  (K)NVDDA LKNPN VTSVE HVIVL K(R)  (R)ILGSV GEPIN PEAWE WYWKK(I) |
| **79** | Hypothetical protein | Q8XFP6 | (R)FYPHI FVEG (-)  (K)GQFTD LQDQV IANLF K(L) |
| **80** | 2,3,-cyclic-nucleotide 2-phosphodiesterase precursor | CN16_SALTY | (K)LPVLS AAAPF K(V)  (K)VTVND ITETA R(K)  (K)FPYVN ANIID VK(T)  (K)TQKPL FTPYL IK(E)  (R)NAADL YLYPN TLVVV K(A)  (K)VAVDD IGFAI YQVDL SK(-)  (K)FAGTG DSHIA FASPD ENR (A)  (K)NLTFN GKPVD PNATF LVATN NYR(A) |
| **81** | Aldehyde dehydrogenase B | Q8ZL72 | (K)EGADI LTGGR(R)  (R)ETSAA DVPLA IDHFR(Y)  (R)VFQEE IFGPV LAVTT FK(T)  (K)ELDGE LKEGY YLEPT ILFGK(N) |
| **82** | Glucose 6-phosphate dehydrogenase | Q8Z5xI | (K)TPELN LFK(E)  (R)LDFCN LDVND TPAFS R(L) |
| **83** | Phosphoenol pyruvate carboxy kinase | PPCK_SALTY | (R)DALLE NVTVR(E)  (K)YTDTP AGEAL VSAGP K(L)  (R)LTANQ TQYHF LSGFT AK(L)  (K)LFIEN FEKYT DTPAG EALVS AGPK(L) |
| **84** | ABC superfamily dipeptide transport protein | Q8Z299 | (R)LVSFI TPDAS VR(Y)  (R)ELNAD DVVFS FDR(Q)  (K)AGTPE KVDLN PVGTG PFQLV QYQK(D) |
| **85** | Phosphoenol pyruvate carboxykinase | PPCK_SALTY | (K)GLVTH QLSGK(R)  (R)GVLTN LGAVA VDTGI FTGR(S)  (R)VSYPI YHIDN IVKPV SK(A) |
| **86** | Polyribonucleotide nucleotidyl transferase, | Q8Z3I0 | (R)IVDFG AFVAI GGGK(E) |
| **87** | Putative cytoplasmic protein | Q8Z8h1 | (R)TEVDE LTR(A)  (R)DIDAL VEQAR(Q) |
| **88** | Chaperone protein  Prolyl tRNA synthetase | HTPG_SALTY  Q8XEY9 | (K)ALEPF VER(V)  R)ELISN ASDAA DKLR(F)  (R)GLIDS NDLPL NVSR(E)  (K)LASGL YTWLP TGLR(V)  (K)TGDIV DYLVK(A)  (K)TIAEL VEQFN LPIEK(T) |
| **89** | Glyceraldehyde 3-phosphate dehydrogenase | G3PI_SALTY | (K)KVVLT GPSK(D)  (R)VPTPN VSVVD LTVRL EK(A) |
| **90** | Fructose bisphosphate aldolase class I  Hypothetical ORF | Q8XFP7  Q8X7H3 | (K)DADSL LQHR(C)  (K)LTSDN PIDLV R(Y)  (K)LINAV QDVYL DSK(V)  (R)LAGTG YLSIL PVDQG VEHSA GASFA AN (G)  (K)LINAV QDVYL DSK(I) |
| **91** | Glyceraldehyde 3-phosphate dehydrogenase | G3PI_SALTY | (R)FDGTV EVK(D)  (R)GASQN IIPSS TGAAK(A)  (K)LVSWY DNETG YSNK(V)  (R)VPTPN VSVVD LTVRL EK(A) |
| **92** | Malate dehydrogenase | MDH_SALTY | (K)NLVQQ IAK(T)  (R)FFSQP LLLGK(N)  (K)LFGVT TLDII R(S)  (K)IQNAG TEVVE AK(A)  (K)RIQNA GTEVV EAK(A)  (K)VAVLG AAGGI GQALA LLLK(N)  (K)ACVGI ITNPV NTTVA IAAEV LK(K) |
| **93** | Putative oxidoreductase | Q8Z3Q5 | (K)AAILN YSR(G)  (R)KALVT GGDSG IGR(A)  (R)EALGG LDILA LVAGK(Q) |
| **94** | Succinyl CoA synthase alpha chain | Q828C5 | (K)VKLDE AGVR(M)  (K)DSILE AIDAG IK(L) |
| **95** | Hypothetical protein slsA | Q9Z622 | (K)VFAVI DASGT YSK(M) |
| **96** | Iron transport protein, periplasmic binding protein | Q9XCS2 | (R)KVIDT IK(K)  (R)AQGAQ LILAN GLNLE R(W)  (K)NVAGD AAEVS SITKP GAEIH EYQPT PGDIK(R)  (R)ESGAH YGGVL YVDSL SAADG PVPTY LDLLR(V) |
| **97** | Glutamate aspartate periplasmic binding protein precursor | GLTI_SALTY | (K)QAAFS DTIFV VGTR(L)  (R)ESSVP FSYYD NQQK(V)  (K)VVGYS QDYSN AIVEA VKK(K)  (R)IPLLQ NGTFD FECGS TTNNL ER(Q) |
| **98** | Glutamate aspartate periplasmic binding protein precursor | GLTI_SALTY | (K)QAAFS DTIFV VGTR(L)  (K)AVVVT SGTTS EILLH K(L)  (R)ESSVP FSYYD NQQK(V)  (K)VVGYS QDYSN AIVEA VK(K  (K)VVGYS QDYSN AIVEA VKK(K) |
| **99** | Glutamate aspartate periplasmic binding protein precursor | GLTI_SALTY | (K)LNKPD LQVK(L)  (K)KLNKP DLQVK(L)  (K)QAAFS DTIFV VGTR(L)  (K)AVVVT SGTTS EILLH K(L)  (R)ESSVP FSYYD NQQK(V)  (R)QKQAA FSDTI FVVGT R(L)  (K)QAAFS DTIFV VGTRL LTK(K)  (K)AVVVT SGTTS EILLH KLNEE QK(M) |
| **100** | D-ribose binding periplasmic protein precursor | RBSB_SALTY | (K)IPVIT LDR(Q)  (K)VIELQ GIAGT SAAR(E) |
| **101** | Hypothetical protein | YGAT_SALTY | (K)ANTDD LAQR(H)  (R)YIDQF VQPK(D)  (R)HTGFT LAPSA QSPR(L)  (K)FLLIN NLFWL HGR(D) |
| **102** | Superoxide dismutase [Mn ] | SODM_SALTY | (K)LDQVP ADK(K)  (R)DFGSV DNFK(A)  (K)KGTTL QGDLK(A)  (R)FGSGW AWLVL K(G)  (R)NNAGG HANHS LFWK(G)  (-)SYTLP SLPYA ADALE PHFDK(Q)  (K)HHQTY VNNA AALEN LPEFA SLPVE ELITK(L) |
| **103** | Ribosome recycling factor | RRF_SALTY | (R)VPLPP LTEER(R)  (R)ASPSL LDGIV VEYYG TPTPL R(Q) |
| **104** | Peptide chain release factor 3 | RF3_SALTY | (K)FTGIP NFAPE LFR(R) |
| **105** | Glutamine binding periplasmic protein | P74886 | (K)SGTGS VDYAK(A)  (R)ENGTY NEIYK(K)  (R)ENGTY NEIYK K(W)  (K)YVGFD VDLWD AIAK(E)  (K)LVVAT DTAFV PFEFK(Q) |
| **106** | 50S ribosomal protein  Two component sensor histidine kinase | RL9_SALTY  Q8YSX3 | (R)DIADA VTAAG VDVAK(S)  (R) LNALEW EGISK (G) |
| **107** | 2,3, phosphoglycerate dependent bisphosphoglycerate | GPMA_SALTY | (K)ELPLT ESLAL TIDR(V)  (K)LSEKE LPLTE SLALT IDR(V) |
| **108** | 2,3, phosphoglycerate dependent bisphosphoglycerate  Triosephosphate isomerase | GPMA_SALTY  TPIS_SALTY | (K)ELPIT ESLAL TIDR(V)  (K)EQGLT PVLCI GETEA ENEAG KTEEV CAR(Q) |
| **109** | Enoyl acil carrier protein reductase | FABI_SALTY | (R)ILVTG VASK(L)  (K)FDGFV HSIGF APGDQ LDGDY VNAVT R(L) |
| **110** | Superoxide dismutase | SODF_SALTY | (K)AQFTD AAIK(N)  (K)LADAI AASFG SFAEF K(A) |
| **111** | DNA protection during starvation protein | Q8XF78 | (K)ASNLLYTR(N)  (R)YAVVA NDVR(K)  (R)YVVA NDVRK(A)  (R)QVIQF IDLSL ITK(Q) |
| **112** | Biotin carboxyl carrier protein | Q8XGD9 | AILVES GQPVE FDEPLVVIE |
| **113** | 2,5, diketo D-gluconic acid reductase A | DKGA_SALTY | (R)EELFI TTK(L) |
| **114** | DNA protection during starvation protein | Q8XF78 | (R)YAVVA NDVRK(A)  (R)AVQLG GVALG TTQVI NSK(T)  (R)DLDKF LWFIE SNIE(-)  (K)AIGEA KDEDT ADIFT AASR(D)  (K)ATVEL LNRQV IQFID LSLIT K(Q) |
| **115** | Pyruvate kinase II | Q8ZNW0 | (K)VIAAG ANVVR(M)  (R)TLNLT ALYR(G)  (R)GDLGV EIGDP ELVGI QK(A)  (K)VFLNI GDKFL LDANL GK(G)  (K)GLPAD VVPGD ILLLD DGRVQ LK(V) |
| **116** | Uridine phosphorylase | UDP_SALTY | (R)IGTTG AIQPH INVGD VLVTT ASVR(L) |
| **117** | Glutamine binding periplasmic protein precursor | P74886 | (K)SGTGS VDYAK(A)  (K)NIDLA LAGIT ITDER(K)  (K)LVVAT DTAFV PFEFK(Q)  (K)AVGES LEAQQ YGVAF PK(G) |
| **118** | Glucose 1-phosphate uridylyltransferase | Q8XEL2 | (R)HNSLG AEFK(A)  (K)ADVAP SNLAI VGR(Y) |
| **119** | Peptidyl prolyl cis-trans isomerase B | Q8XFG8 | (K)EAIKN EANNG LK(N) |
| **120** | Putative aldehyde dehydrogenase | Q8ZPC9 | (R)VITGG SQTEG K(G)  (K)DIFPP GVLNV LFGR(G)  (K)APVIV FDDAD LDAVA QGVR(T)  (K)LAVLA KDIFP PGVLN VLFGR(G) |
| **121** | Superoxide dimutase | SODF_SALTY | (K)LADAI AASFG SFAEF K(A) |
| **122** | Hypothetical protein | YAJQ_SALTY | (K)QGIES AVQK(K)  (R)GGDLG QPFQF K(N)  (K)VLSES DFQVN QLLDI LR(A) |
| **123** | Phosphoenol pyruvate protein phosphotransferase | PT1_SALTY | (K)VLGFI TDAGG R(T)  (K)ALLLK EDEIV IDR(K)  (K)AVQEQ VATEK AELAK(L) |
| **124** | Nucleoside diphosphate kinase | NDK_SALTY | (K)NVIGS IFAR(F)  (R)DLLGA TNPAN ALAGT LR(A)  (R)HRDLL GATNP ANALA GTLR(A) |
| **125** | Peptidyl prolyl cis-trans isomerase B | PPIB_ECOLI | (K)TFDDK APETV K(N) |
| **126** | Cold shock protein | CSPC_ECOLI | (K)GFGFI TPADG SK(D) |
| **127** | Hypothetical protein | Q8XGB9 | (R)HATLP VLVVR(-) |
| **128** | Hypothetical protein | YJJK_ECOLI | (K)IGVLG LNGAG K(S)  (K)NISLS FFPGA K(I)  (R)ESIEE AVSEV VNALK(R) |
| **129** | Periplasmic oligopeptide binding protein precursor | OPPA_SALTY | (K)LALDR DIIVN K(V) |
| **130** | ATP dependent protease proteolytic subunit | CLPP_SALTY | (R)SFDIY SR(L)  (R)FLSAP EAVEY GLVDS ILTHRN (-) |
| **131** | ABC transporter ATP binding protein | Q89Q97 | (R)ALLDA VPRAR(A) |
| **132** | Putative serine protein kinase | Q8ZPW2 | (R)LLCEW YLR(V)  (K)QILYL LGPVG GGK(S)  (R)VPIYV LSANG ER(S)  (R)VYDGE SLKDT DPK(A) |
| **133** | Glutaminyl tRNA symthetase | SYQ_SALTY | (K)GLAYV DELTP EQIR(E) |
| **134** | Fumarate reductase flavoprotein subunit | Q8XFL5 | (R)LGSNS LAELV VFGR(L) |
| **135** | Putative alpha amylase | Q8ZPF0 | (R)HVLVE IAQR(I) |
| **136** | Citrate synthase | CISY_SALTY | (R)QLYTG YDKR(D)  (K)GTLGQ DVIDI R(S)  (K)YSIGQ PFVYP R(N)  (K)ITFID GDEGI LLHR(G)  (K)LYPNV DFYSG IILK(A) |
| **137** | Putative aldehyde dehydrogenase | Q8Z747 | (K)LGNAV SSLK(M)  (R)GQTVG DVLTG HEK(V)  (K)GYYFA PTLLA DAK(Q)  (K)DIFPP GVLNV LFGR(G)  (K)LADSI EQNAL EFAR(L)  (K)LAPAL AAGNC VVIKP SEITP LTALK(L) |
| **138** | Dihydrolipoamide dehydrogenase | Q8Z9E8 | (K)TQVVV LGAGP AGYSA AFR(C)  (K)VIPSI AYTEP EVAWV GLTEK(E)  (K)VIPSI AYTEP EVAWV GLTEK EAK(E)  (K)TVINF DNAII AAGSR PIQLP FIPHE DPR(V) |
| **139** | Putative NAD dependent aldehyde dehydrogenase | Q8ZJZ3 | (K)DALET LTR(Q)  (R)VQGAA LTGSE K(A)  (R)VLAPN LAAGN PVLAK(H)  (K)NGATL HVGGK PLESK(G)  (K)LADLI DSRVE ELAK(I)  (R)ELSDL GIKEF VNQK(L)  (K)GNFFE PTILT HITR(D)  (K)IADQF LSQFT EAFR(K)  (K)IADQF LSQFT EAFRK(V) |
| **140** | Succinylarginine dihydrolase  Glutathione oxidoreductase | Q8ZPU9  Q8ZLD4 | (R)EALDV LTR(L)  (R)ALEAP VTEAL LR(A)  (R)LTAAD LADPL LLR(E)  (K)NADGS LTLEL EDGR(S) |
| **141** | Succinyl ornithine transaminase | ARGM_SALTY | (R)HDAFI ER(L) |
| **142** | Glutamate dehydrogenase | Q8Z691 | (R)GVFVS GLEAA R(R)  (R)VAVQG FGNVG SEAAR(L)  (R)VDPFS LSEGE LER(L) |
| **143** | Acetate kinase | ACKA_SALTY | (R)YVEDN YATK(E)  (K)LGVLG FEVDH ER(N)  (K)EGTRP AVVIP TNEEL VIAQD ASR(L) |
| **144** | 2-amino 2-ketobutyrate CoA ligase  Carbamoyl phosphate synthase small chain  Alcohol dehydrogenase class III | KBL_SALTY  CARA_SALTY  Q8ZPA8 | (R)VDIIT GTLGK(A)  (K)ALGGA SGGYT AAR(K)  (K)EGIYV TGFFY PVVPK(G)  (R)LTVVP AQTSA EEVLK(M)  (R)AAVAF GPGQP LK(I) |
| **145** | Maltose binding periplasmic protein precursor  Aspartate aminotransferase | MALE_SALTY  AAT_SALTY | (R)TAVIN AASGR(Q)  (K)LYPFT WDAVR(Y)  (K)VNYGV TLLPT FK(G)  (K)NYLGI DGIPE FAR(C)  (R)ANYSN PPAHG ASIVA TILSN DALR(A) |
| **146** | Histidine ammonia-lyase | HUTH_SALTY | (R)IADED LQNLQ R(S)  (K)EGLAL LNGTQ ASTAF ALR(G)  (R)LSVIE ALIAL VNAGV YPLIP AK(G) |
| **147** | 3-oxoacyl [acyl carrier protein] synthase II | Q8Z7J4 | (K)ASTLPGVGGFGAAR(A)  (R)IGAAIGSGIGGLGLIECNHSSLVK(G)  (K)ALLAGQSGISLIDHFDTSAYATK(F)  (R)DAAIEPAQIGYVNAHGTSTPAGDKAETQAVK(S) |
| **148** | Phosphoserine aminotransferase | SERC_SALTY | (R)NDVAQ ANR(S)  (R)YGVIY AGAQK(N)  (R)DLLNI PSNYK(V)  (K)NIGPA GLTLV IVR(E)  (K)AELLY GVIDN SDFYR NDVAQ ANR(S) |
| **149** | Leucine isoleucine threonine binding protein precursor | LIVJ_SALTY | (R)TTGLD SDQGP TAAK(Y)  (K)NYDQV PANKP IVDAI K(A) |
| **150** | Succinyl CoA synthase beta chain | SUCC_SALTY | (R)LEGNN AELGA K(K)  (K)ELYLG AVVDR(S)  (K)VAEET PHLIH K(V)  (R)DLALI EINPL VITK(Q)  (K)SLTDA AQQVV AAVEG K(-)  (K)IILSD DNVKA VLVNI FGGIV R(C) |
| **151** | ATP synthase alpha chain | Q8XG95 | (K)TALAI DAIIN QR(D) |
| **152** | ADP-L-glycero-d-manno-heptose-6-epimerase | RFAD_SALTY | (R)TSDFI ESR(E)  (R)YQAFT QADLT NLR(N) |
| **153** | Transcriptional termination factor Rho | RHO_SALTY | (R)VLDLA SPIGR(G)  (R)VFPAI DYNR(S)  (K)DVIIL LDSIT R(L) |
| **154** | Biotin carboxylase | Q8XF58 | (K)NALQE LIIDG IK(T)  (K)VVEEA PAPGI TPELR(R) |
| **155** | Succinyl glutamic semiladehyde dehydrogenase | Q8Z6G1 | (R)AGLPA GVLNL VQGGR(E) |
| **156** | Glutaredoxin 2 | G8XG23 | (K)KIGDD LR(L)  (K)SAFDE FSTPA AR(Q)  (K)VNGYV NQLLL PR(F)  (K)LIVQP NAVNG ELSED DIHLF PLLR(N) |
| **157** | 2-methylcitrate dehydratase  Threonine synthase | PRPD_SALTY  Q8Z9R6 | (R)AIGNA ITVEF TDGSR(F)  (K)LLGPI VPGTV VPNGA R(V)  (K)QAFDD EELKT ALGLN SANSI NISR(L) |
| **158** | Arginyl tRNA synthetase | SYR_SALTY | (K)VEIAG PGFIN IFLEP AFLAE QVQQA LASDR(L) |
| **159** | Oligopeptide binding protein precursor | OPPA_SALTY | (K)DPLDN IYVK(N)  (K)ALDDH TFEVT LSEPV PYFYK(L)  (K)AEQQL DKDSA IVPVY YYVNA R(L) |
| **160** | Ribose phosphate pyrophosphokinase | KPRS_SALTY | (R)LYTSL GDAAV GR(F)  (K)VVADF LSSVG VDR(V)  (R)ITAVI PYFGY AR(Q) |
| **161** | Putative universal stress protein | Q8ZP84 | (R)YYLEA GVPIE IK(V) |
| **162** | Lysyl tRNA synthetase | SYK1_SALTY | (R)YLDLI SNDES R(N) |
| **163** | Lysyl tRNA synthetase | SYK1_SALTY | (R)LVVGG FER(V)  (R)FEFFI GGR(E)  (R)YLDLI SNDES R(N) |
| **164** | D-mannonate hydrolase | GI/16761913 | (R)AIIPVADEVGVR(V) |
| **165** | Threonine synthase | THRC_ECOLI | (R)FIAAT NVNDT VPR(F) |
| **166** | Putative nucleotide binding protein | Q8ZA36 | (K)NQVLP LILTG PK(E) |
| **167** | Survival protein | Q8XEV3 | (R)ITVLP QEVDA LAK(Q) |
| **168** | 2-methyl citrate synthase | PRPC_SALTY | (R)VIAGT GSDVY SAIIG AIGAL RGPK(H) |
| **169** | Pyruvate dehydrogenase | Q8Z831 | (R)AFSID GPVLV DVVVA K(E) |
| **170** | 2,3,4,5 tetrahydropyridine 2,6-dicarboxylate N-succinyl transferase | DAPD_KLEPN | (K)QVISLLDSGALR(V)  (R)ADITPANVDTVTR(E)  (R)RADITPANVDTVTR(E) |
| **171** | Putative ABC superfamily transport protein | Q8ZNM8 | (R)IAVEG LDAK(K)  (K)LAASA EFIER ADALP AFEK(A) |
| **172** | Dihydrolipoamide succinyl transferase component | Q8XFE6 | (R)GLVTP VLR(D)  (K)APAVE PAAQP ALGAR(G) |
| **173** | Glycyl tRNA synthetase beta chain | SYGB_SALTY | (K)VIPAT ILGIQ SDR(V)  (R)LADAE FFFNT DR(K)  (K)TFLVE IGTEE LPPK(A)  (K)NLALD LQTLT EEAVR(L) |
| **174** | Transketolase | Q8Z3V6 | (R)TEEQL ANIAR(G)  (R)QDGPT ALILS R(Q)  (R)GIDGH DADAI KR(A)  (K)YGVER QDGPT ALILS R(Q)  (K)YAPFE IPSEI YAQWD AK(E) |
| **175** | Acetyl CoA synthase | Q8ZKF6 | (K)HAIPA NIADR(C)  (R)FANTL LDLGI K(K)  (R)RDEDG YYWIT GR(V)  (R)IGAVH SVIFG GFSPE AVAGR(I)  (K)EIGPL ATPDV LHWID SLPK(T) |
| **176** | Succinate dehydrogenase flavoproteinsubunit | DHSA_SALTY | (R)LPGIL ELSR(T)  (R)LGGNS LLDLV VFGR(A) |
| **177** | Multifunctional folypolyglutamate synthase | Q8Z501 | (R)LDILK PAPFV FTVAG TNGK(G) |
| **178** | Putative cytoplasmic protein | Q8ZPB1 | (R)EVFNA FPDSE FLLR(Q) |
| **179** | Putative aldehyde dehydrogenase  Glutamyl-tRNA synthetase | Q8Z747  SYE_SALTY | (K)LGNAV SSLK(M)  (K)GYYFA PTLLA DAK(Q)  (K)LADSI EQNAL EFAR(L)  (R)GIYDA LVEKL GNAVS SLK(M)  (K)GYYFA PTLLA DAKQE DAIVQ R(E)  (R)TALYS WLFAR(H)  (R)DDGYL PEALL NYLVR(L)  (R)GPIEF SNQEL DDLII R(R)  (R)GPIEF SNQEL DDLII RR(T) |
| **180** | Glutamate dehydrogenase | Q8Z691 | (R)GVFVS GLEAA R(R)  (R)VAVQC FGNVG SEAAR(L)  (K)QIAGF PGAET IASDA FWR(L)  (K)LVLEG ANGPT YPDAD DVLAS R(G)  (R)VAVQG FGNVG SEAAR LFAGA GAR(V) |
| **181** | Tyrosyl tRNA synthetase | Q8XG70 | (R)AQYVL AEQVT R(L) |
| **182** | Probable aminotransferase | Q8Z3L9 | (K)SLGAL SATAK(S)  (R)VLVAG TLNNA K(T)  (R)EVIDY FKEHV NPGFL EYR(K)  (K)TGDEI AAVIL EPIQC EGGVI LPPQG YLTEVR(KI)  (K)KTGDE IAAVI LEPIQ GEGGV ILPPQ GYLTEVR(K) |
| **183** | FliY putative periplasmic binding transport protein | Q9ZNM2 | (K)GTLAV TGDAF SR(Q)  (R)VGRID AILVD R(L)  (K)VGVGL GTNYE EWLR(Q)  (K)RIDVV INQVT ISDVR(K)  (K)YDFST PYTVS GIQAL VK(K) |
| **184** | Hypothetical oxidoreductase | Q8XGU5 | (R)EAALT YAR(Y)  (R)IILVT GASDG IGR(E)  (K)SDAGS LVFTS SSVGR(Q) |
| **185** | Putative arginine deaminase | Q8Z125 | (K)AGITV LPIPG DELGR(G) |
| **186** | Serine endoprotease | Q8Z3E6 | (R)GNENI YLLLR(-) |
| **187** | Glucose 6-phosphate isomerase | G6PI_SALTY | (K)LLSNF FAQTE ALAFG K(S) |
| **188** | Hypothetical protein | YBGI_SALTY | (K)IVTGV TASQA LLDEA VR(L) |
| **189** | Soluble pyridine nucleotide transhydrogenase | STHA_SALTY | (R)SSFAD ILNHA DNVIN QQTR(M) |
| **190** | Iron transport protein periplasmic binding protein | Q9XCS2 | (R)VTTET IVNGI NDGLR(S) |
| **191** | UTP Glucose 1 –phosphate uridylyl transferase | GALU_SALTY | (K)ADVAP SNLAI VGR(Y) |
| **192** | Aldehyde dehydrogenase B | Q8ZL72 | (K)EGYYL EPTIL FGK(N)  (R)ETSAA DVPLA IDHFR(Y)  (R)VFQEE IFGPV LAVTT FK(T) |
| **193** | Succinate semiladehyde dehydrogenase | Q8ZMM2 | (R)LYVQD GVYDR(F)  (K)EETFG PLAPL FR(F)  (K)VAKEE TFGPL APLFR(F)  (R)AGIPA GVFNV VTGSA GDIGG ELTSN PLVR(K)  (K)VSLEL GGNAP FIVFD DADLD KAVEG ALASK(F) |
| **194** | Glutamine synthetase | GLNA_SALTY | (R)IPVVA SPK(A)  (K)RAEDY LR(A)  (R)SASIR IPVVA SPK(A)  (K)GGYFP VPPVD SAQDI R(S) |
| **195** | Adenylate kinase | KAD_SALTY | (K)VDGTQ AVADV R(A)  (R)NGFLL DGFPR(T)  (K)LVTDE LVIAL VK(E) |
| **196** | 2,3 phosphoglycerate dependent phosphoglycerate | GPMA_SALTY | (R)GFAVTPPELTK(D)  (R)FTGWYDVDLSEK(G)  (K)ELPLTESLALTIDR(V)  (R)AIHTLWNVLDELDQAWLPVEK(S) |
| **197** | Periplasmic trehalose precursor | TREA_SALTY | (R)VVKLE DGSVL NR(Y)  (K)SNPNR PATEI YR(D)  (K)VAAAA QAHLL QPGGL ATTSV K(S)  (K)NVEKW DSLLP LPESY VVPGG R(F) |
| **198** | Periplasmic oligopeptide binding protein precursor | OPPA_SALTY | (K)DPLDN IYVK(N)  (K)ALDDH TFEVT LSEPV PYFYK(L)  (R)LADPN TASPY ASYLQ YGHIA NIDDI IAGK(K) |
| **199** | Serine hydroxymethyl transferase | GLYA_SALTY | (K)VLDIC AR(F)  (K)SPFVT SGIR(I)  (K)NSVPN DPKSP FVTSG IR(I) |
| **200** | Glutamine binding periplasmic protein precursor | P74886 | (K)AIDFS DGYYK(S)  (R)ENGTY NEIYK K(W)  (K)YVGFD VDLWD AIAK(E)  (K)LVVAT DTAFV PFEFK(Q)  (K)AVGES LEAQQ YGVAF PK(G)  (R)ADAVL HDTPN ILYFI K(T) |
| **201** | Glyceraldehyde 3-phosphate dehydrogenase A | G3PI_SALTY | (R)VPTPN VSVVD LTVRL EK(A) |
| **202** | Quinone oxidoreductase | QOR_SALTY | (K)LIGTV GSAQK(A)  (K)AIGIN FIDTY IR(S) |
| **203** | Bifunctional purine biosynthesis protein | PUR9_SALTY | (R)TLNLN FIK(K)  (K)AGIIE FAQAL SAR(G) |
| **204** | CTP synthase | PYRG_SALTY | (K)LIDSQ DVETR(G)  (K)GIAAA SLAAI LEAR(G) |
| **205** | Phosphoglycerate kinase  Recombinase A | PGK_SALTY  RECA_SALTY | (R)ASPLT IELAL K(Q)  (R)VATEF SETAP ATLK(S)  (K)SLYEA DLVDE AKR(L)  (K)IADQL IVGGG IANTF VAAQG HSVGK(S)  (K)LVKDY LDGVD VAEGE LVVLE NVR(F)  (K)EGDNV VGSET R(V) |
| **206** | Transaldolase A | TALA_SALTY | (R)VSTEV DAR(L)  (K)LAVNF GAEIL K(S)  (K)QFTTV VADSG DIESI R(H) |
| **207** | Peroxidase/catalase HPI | CATA_SALTY | (R)ADLVF GSNSV LR(A)  (R)FLNDP QAFNE AFAR(A) |
| **208** | ATP synthase beta chain | ATPB_ECOLI | (R)DVLLF VDNIY R(Y)  (R)YTLAG TEVSA LLGR(M) |
| **209** | Histidyl tRNA synthetase | SYH_SALTY | (R)LPIVE QTPLF K(R) |
| **210** | Sulfate binding protein | SUBI_SALTY | (K)NFYRP R(D)  (K)KYDDA FPK(L)  (K)QETGD NVVIR(Q)  (K)YLYSP EGQEI AAK(N)  (R)LPDNS APYTS TIVFL VR(K)  (K)RLPDN APYTS TIVFL VR(K)  (K)DKFEI VTPSE SILAE PTVSV VDK(V)  (K)QATSV INGIE ADVVT LALAY DVDAI AER(G) |
| **211** | Thiosulfate binding protein | CYSP_SALTY | (K)QALAI LQGLK(A)  (K)DNGGD KLTIK(Q)  (K)QYEAQ GFEVV IPK(T)  (R)GLGDV LISFE SEVNN IR(K)  (R)GLGDV LISFE SEVNN IRK(Q) |
| **212** | Cysteine synthase A | CYSK_SALTY | (K)AEEIV ASDPQ K(Y)  (K)LQEDE SFTNK(N)  (K)ALGAN LVLTE GAK(G)  (K)IQGIG AGFIP GNLDL K(L)  (R)YLSTA LFADL FTEK(E)  (K)LIDKV VGITN EEAIS TAR(R)  (R)GVLKP GVELV EPTSG NTGIA LAYVA AA(R) |
| **213** | DNA directed RNA polymerase alpha chain | RPOA_ECOLI | (R)TEVEL LK(T)  (K)SLTEI KDVLA SR(G)  (K)EEKPE FDPIL LRPVD DLELT VR(S) |
| **214** | Adenylsuccinate lyase | Q8ZPZ6 | (R)AVTLV DELK(-)  (K)EVPAF AADAN GYLTD LVANF NEEDA AR(I) |
